# A Rare Case of Cyclophosphamide-Induced Posterior Reversible Encephalopathy Syndrome in a Patient with Anti-GBM Vasculitis, and Review of Current Literature

**DOI:** 10.1155/2019/2418597

**Published:** 2019-09-29

**Authors:** Taha Nisar, Abdul R. Alchaki, Erin Feinstein

**Affiliations:** Rutgers New Jersey Medical School, Newark, USA

## Abstract

Posterior reversible encephalopathy syndrome (PRES) is a clinical syndrome of headache, altered mental status, and seizures with reversible mainly posterior leukoencephalopathy on neuroimaging. Precipitating factors for PRES are multifactorial and include autoregulatory failure due to changes in blood pressure, metabolic derangements, and cytotoxic medications. We report the second case of cyclophosphamide-induced PRES in a patient with anti-glomerular basement membrane (Anti-GBM) positive vasculitis. In the acute setting, PRES can be challenging to distinguish from cerebral venous sinus thrombosis or cerebral vasculitis based on clinical presentation. Neuroimaging with magnetic resonance imaging (MRI) of the brain along with a vessel imaging, can help reach the diagnosis.

## 1. Introduction

Posterior reversible encephalopathy syndrome (PRES) is a clinical syndrome of headache, altered mental status, and seizures with reversible mainly posterior leukoencephalopathy on neuroimaging [1]. Precipitating factors for PRES are multifactorial and include autoregulatory failure due to changes in blood pressure, metabolic derangements, and cytotoxic medications [2]. As the name implies, PRES is generally reversible and resolves by treating the underlying cause [1–3]. The use of immunosuppressants is a known risk factor for the development of PRES [4]. Among cytotoxic medications, cyclosporine is the best reported to cause PRES, but many other medications have also been reported to have PRES as a complication [2, 5]. We report a rare case of a patient who was given cyclophosphamide for the treatment of Anti-glomerular basement membrane (Anti-GBM) positive vasculitis, and developed PRES.

## 2. Case Report

**Figure 1:**
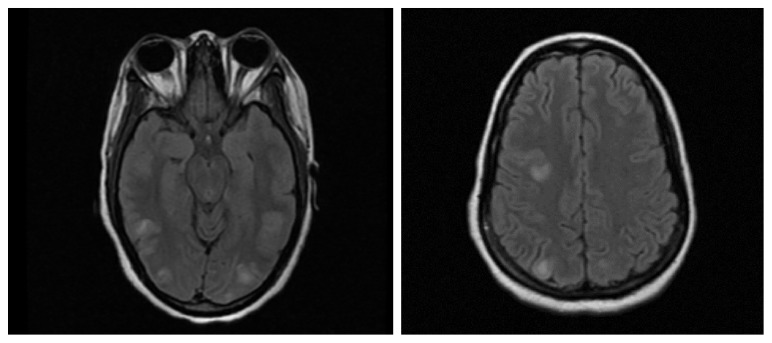
MRI brain without contrast showing areas of cortical and subcortical white matter hyperintensities likely secondary to posterior reversal encephalopathy syndrome on the FLAIR sequence. *Abbreviation:* MRI, Magnetic Resonance Imaging; FLAIR, Fluid attenuation inversion recovery.

**Figure 2:**
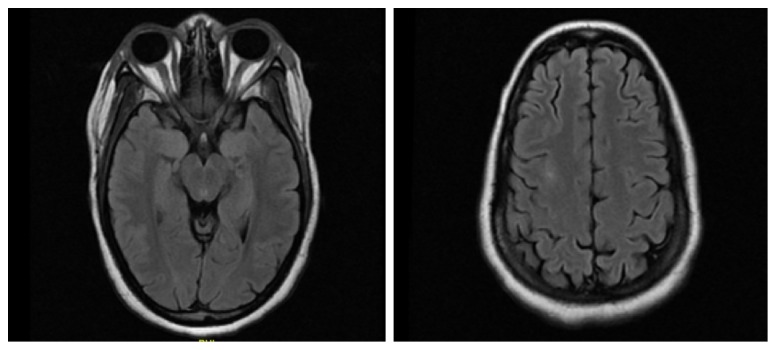
MRI brain without contrast five days later showing resolution of previously seen areas of cortical and subcortical white matter hyperintensities on the FLAIR sequence. *Abbreviation:* MRI, Magnetic Resonance Imaging; FLAIR, Fluid attenuation inversion recovery.

29-year-old African American woman presented to the emergency department for three weeks of nausea, vomiting, and diarrhea; and five days of anuria. She was found to be uremic with renal failure (Creatinine: 5.5 mg/dl, blood urea nitrogen: 34 mg/dl, creatinine clearance: 6 ml/min). Renal biopsy showed necrotizing glomerulonephritis (Immunoglobulin G, perinuclear anti-neutrophil cytoplasmic antibodies, anti-glomerular basement membrane positive). She was treated with intravenous methylprednisolone started on day 5 of hospitalization (1 g intravenous daily for three days followed by 24 mg twice a day), plasmapheresis on day 8 of hospitalization, and cyclophosphamide (induced at a dose of 510 mg because of renal failure and plasmapheresis) on day 9 of hospitalization. Two days after cyclophosphamide induction, the patient became encephalopathic and developed clinical seizures consisting of left gaze deviation, left neck tonic deviation, left eye blinking, left arm flexion with rhythmic jerking in conjunction with the head. Fosphenytoin (loaded with 20 mg/kg followed by phenytoin maintenance at 100 mg three times a day) was initiated for seizures, and the patient was intubated and sedated on propofol drip. Blood pressure remained normotensive throughout hospitalization (Mean: 122.5/77, Median: 122/78 over a range of ten days of the event). The initial working diagnosis was cerebral vasculitis versus cerebral venous thrombosis. Magnetic resonance angiography (MRA) showed clear patent vessels, with no signs of venous thrombosis. Magnetic resonance imaging (MRI) revealed areas of cortical and subcortical white matter T2 Fluid-attenuated inversion recovery (FLAIR) hyperintensities in bilateral occipital-parietal and the right frontal region, as shown in Figure 1. Continuous electroencephalogram monitoring showed diffuse interictal slowing. Seizures and encephalopathy in the setting of chemotherapy treatment with the radiographic findings were most consistent with PRES. Patient self-extubated three days later and was oriented to person, place, and time with no clear neurologic deficits on examination. Repeat MRI done six days after the initial scan showed resolution of previously seen areas of T2 FLAIR, white matter hyperintensities as shown in Figure 2. She was discharged home on carbamazepine 400 mg BID and taper of phenytoin (100 mg every third day followed by stopping), and a steroid taper (methylprednisolone 16 mg BID for two weeks with a plan to taper by 4 mg BID every two weeks if tolerated).

## 3. Discussion

First described in 1996, PRES is a clinical syndrome with seizures, headache, altered mental status, and visual disturbances [1]. Data from case series suggest generalized tonic-clonic seizure is the most common presentation, followed by encephalopathy and headache [6, 7]. The exact incidence of PRES is unknown, though it is being increasingly reported [2]. PRES has a female preponderance and usually afflicts middle-aged patients though the range of patient's age can range from 4 years to 90 years [1–4]. Neuroimaging shows symmetrically posterior leukoencephalopathy that improves with time [1, 3]. The classic description is the parietooccipital pattern of vasogenic edema in predominantly subcortical areas, though cortical involvement is also common [1, 8]. Involvement of parietooccipital lobe (≥98%) is more common than frontal lobe (around 70%), though temporal lobe (around 65%), cerebellar (30–53%), basal ganglia (630%), and midbrain involvement (630%) have also been reported [8]. The findings of PRES are appreciated on MRI on T2 sequences which show punctate or confluent areas of increased signal [8, 9]. The MRI findings usually resolve in 7–14 days [3]. Brain biopsy in PRES reveals edema in white matter with no evidence of vessel wall damage or infarction [10].

Causes of PRES are multifactorial, with a common pathway of autoregulatory failure or endothelial damage, that lead to capillary leakage with vasogenic edema [1, 2, 11]. Uncontrolled hypertension, chemotherapeutic agents, eclampsia, and hypomagnesemia are some factors associated with the development of PRES [1, 4, 12].

The exact mechanism of PRES is unknown; there is evidence to support the notion that autoregulatory failure leading to cerebral hyperperfusion or hypoperfusion is thought to be causative [4]. Schwartz et al. proposed an increase in perfusion using Single Photon Emission Computed Tomography (SPECT) scans, while Bartynski et al. showed a decrease in perfusion on relative cerebral blood volume (rCBV) color maps [13, 14]. Acute and rapid elevation of BP above the limit of autoregulation is a common cause of PRES and is associated with 75% of the cases [1, 11]. Rapid elevation of BP is thought to overwhelm the autoregulatory mechanisms, causing arteriolar dilation and break down of the blood-brain barrier, and leakage of plasma into brain parenchyma [15].

Our patient was normotensive and developed PRES after receiving one dose of cyclophosphamide in addition to three cycles of plasmapheresis and six days of intravenous methylprednisolone. The use of immunosuppressants is a known risk factor for the development of PRES [4]. PRES as a complication of immunosuppression has most often been reported with cyclosporine and tacrolimus, though interferon-alpha, intrathecal methotrexate, bevacizumab, gemcitabine, and sirolimus have also been reported [5, 16–24]. immunosuppressive therapies are thought to have a direct toxic effect on endothelium causing arteriolar and capillary damage with disruption of the blood–brain barrier and subsequent vasogenic edema, causing PRES [1, 22].

There is a paucity of case reports identifying cyclophosphamide as a trigger for PRES in the rheumatologic population. Many of these cases developed PRES in the setting of acute renal failure when treated with cyclophosphamide for a rheumatological condition, similar to our case [25–30]. These cases are summarized in Table 1. Chang-Hoon Lee reported a case of a 42-year-old woman with systemic lupus erythematosus (SLE) who presented with renal failure secondary to glomerulonephritis and developed PRES after two cycles of cyclophosphamide [30]. Jabrane et al. reported a case of a 16-year-old girl with SLE and lupus nephritis who presented with renal failure and developed sudden onset of a headache, blurring of vision, followed by three episodes of generalized seizures after being treated with IV cyclophosphamide pulse therapy at 300 mg/m^2^ of the body surface [28]. Zekić et al. reported a case of an 18-year-old girl with of SLE who presented with renal failure due to lupus nephritis and acute arthritis and developed PRES after administration of the second dose of cyclophosphamide [25]. Jayaweera et al. reported a case of a 33-year-old woman who presented with renal failure due to lupus nephritis who developed PRES after the second dose of cyclophosphamide [27]. Abenza-Abildua reported another case of PRES in a 27-year-old man with high blood pressure (HBP) and glomerulonephritis caused by anti-GBM disease treated with cyclophosphamide [29]. Di Pan reported PRES in a 22-year-old woman with a history of Sjogren's syndrome who had an acute respiratory failure from diffuse alveolar hemorrhage concurrent with renal failure from glomerulonephritis, who was treated with cyclophosphamide on day 2, and developed seizures on day 5, with findings concerning for PRES on neuroimaging [26]. All of these case reports are young individuals with an underlying rheumatological condition causing renal failure who developed PRES while receiving cyclophosphamide for their underlying rheumatologic condition. Majority of these cases had SLE with nephritis, however along with Abenza-Abildua et al., our case reports PRES caused by cyclophosphamide use to treat Anti-GBM vasculitis [26]. Our case report provides further insight into the pathophysiology of immunosuppressants causing PRES; specifically in patients with renal failures and Anti-GBM vasculitis undergoing treatment with cyclophosmaphide.

## 4. Conclusion

To our knowledge, this is only the second reported case in the literature associated with the development of PRES due to cyclophosphamide given for (Anti-GBM) vasculitis. There are several other case reports of young individuals with underlying rheumatological conditions developing renal failure and subsequent PRES after receiving cyclophosphamide. In the acute setting, PRES can be difficult to distinguish from cerebral venous sinus thrombosis, cerebral vasculitis based on clinical findings. Neuroimaging with magnetic resonance imaging (MRI) of the brain along with vascular imaging can help reach the diagnosis.

## Figures and Tables

**Table 1:** Case reports of posterior reversible encephalopathy syndrome (PRES) in patients given cyclophosphamide.

Case reports	Demographics	Rheumatological condition	Laboratory investigations	Treatments given	MRI findings
Chang-Hoon Lee et al. [30]	42-year-old female	SLE	Cr: 2.68 mg/dl, BUN: 41.7 mg/dl	Cyclophosphamide, steroids, anticonvulsive and antihypertensive medications	T2 hyperintensities involving the bilateral parieto-occipital lobes, frontal lobes, and basal ganglia
Jabrane et al. [28]	16-year-old female	SLE	BUN: 58 mg/dl, CrCl: 5 ml/min, Proteinuria 2.5 g/day	Cyclophosphamide, steroids	T2 hyperintensities on bilateral occipital lobes
Zekić et al. [25]	18-year-old female	SLE	Proteinuria 2.7 g/day	Cyclophosphamide, steroids	T2 hyperintensities involving the bilateral parieto-occipital lobes and right frontal lobe
Jayaweera et al. [27]	33-year-old female	SLE	Cr: 5.79 mg/dl	Cyclophosphamide, steroids, midazolam, phenytoin, valproate, topiramate	T2 hyperintensities on bilateral occipital lobes
Abenza-Abilua et al. [29]	27-year-old male	Anti-GBM disease	Cr: 8.50 mg/dl, BUN: 149 mg/dl, CrCl: 6 ml/min	Cyclophosphamide, steroids	T2 hyperintensities involving the bilateral parieto-occipital lobes
Di Pan et al. [26]	22-year old female	Sjogren's syndrome	Cr: 1.47 mg/dl, BUN: 41.7 mg/dl	Cyclophosphamide, steroids	T2 hyperintensities involving the bilateral parieto-occipital lobes and cerebellum

MRI, Magnetic Resonance Imaging; SLE, Systemic Lupus Erythematosus; Anti-GBM, Anti-Glomerular Basement Membrane; Cr, Creatinine; BUN, Blood Urea Nitrogen; CrCl, Creatinine Clearance.
